# Na ^+^ /K ^+^ -ATPase Blockade Reversibly Attenuates Proliferation and Metabolic Function in K562 Human Myelogenous Leukemia Cells

**DOI:** 10.64898/2026.09.11.751086

**Published:** 2026-09-17

**Authors:** Simon R Bowden, Eli T Colton, Amber D Norgaard, McKenzie M Haas, Erynn R Dunnigan, Carly J Goven, Grace O Goven, Thomas J Hinnenkamp, Keagan D Walker, Theresa M Ballance, Blaise I Kathol, Elizabeth R Murphy, Kristian M Van Slyke, Rylie J Webb, Ashlin B Schaefbauer, Emily R Biggane, Joseph P Biggane

## Abstract

The Na ^+^ /K ^+^ -ATPase consumes a substantial portion of cellular ATP to maintain essential electrochemical gradients, yet its role in regulating cancer cell proliferation and phenotypic plasticity remains complicated by its dual function as an ion transporter and a scaffolding receptor. Here, we investigate the physiological consequences of pharmacological NKA blockade using digoxin in K562 human chronic myelogenous leukemia cells. Submicromolar digoxin exposure induced a concentration-dependent attenuation of cell proliferation (IC _50_ = 151.0 nM) and a statistically significant depression of metabolic reducing capacity without impairing cell viability. Competitive supplementation with extracellular potassium salts produced a surmountable rightward shift in digoxin sensitivity, confirming that growth inhibition is driven by on-target NKA pump occupancy rather than non-specific interactions. Furthermore, this cytostatic growth attenuation and metabolic depression were found to be fully reversible upon drug clearance. K562 cells are largely refractory to canonical kinase-overdrive stress checkpoints as they harbor constitutive BCR-ABL tyrosine kinase activity alongside non-functional TP53 and a homozygous deletion of CDKN2A (p16 ^INK4a^ ). Consequently, these findings suggest that NKA perturbation attenuates K562 growth primarily through ion dyshomeostasis and secondary active transport constraints rather than scaffold-mediated signaling. Overall, we present a novel model for elucidating the role of the NKA in cancer proliferation and dissecting the mechanistic underpinnings of ion-mediated alterations in cancer cell physiology.

## Full Text Availability

The license terms selected by the author(s) for this preprint version do not permit archiving in PMC. The full text is available from the preprint server.

